# Effect of Proanthocyanidin, Fluoride and Casein Phosphopeptide Amorphous Calcium Phosphate Remineralizing Agents on Microhardness of Demineralized Dentin

**Published:** 2017-03

**Authors:** Zahra Khamverdi, Matin Kordestani, Ali Reza Soltanian

**Affiliations:** 1 Professor, Dental Research Center, Department of Restorative Dentistry, Dental School, Hamadan University of Medical Sciences, Hamadan, Iran; 2 Post Graduate Student, Department of Restorative Dentistry, Dental School, Hamadan University of Medical Sciences, Hamadan, Iran; 3 Professor, Department of Biostatiatics, School of Public Health and Modeling of Noncomunicable Diseases Research Center, Hamadan University of Medical Sciences, Hamadan, Iran

**Keywords:** Dentin, Fluoride, Proanthocyanidin, Remineralization

## Abstract

**Objectives:**

The aim of this study was to evaluate the effect of dentin remineralization using proanthocyanidin (PA), fluoride varnish and casein phosphopeptide amorphous calcium phosphate (CPP-ACP) paste and their various combinations on microhardness of demineralized root dentin.

**Materials and Methods:**

One-hundred and twenty freshly extracted sound human premolars were selected and randomly divided into eight groups for dentin treatment as follows. C: Deionized water (control); PA: 6.5% PA solution; F: fluoride varnish (5% NaF, 22600 ppm fluoride); CP: CCP-ACP; PAF: 6.5% PA + fluoride varnish; PACP: 6.5% PA + CCP-ACP; FCP: fluoride varnish + CCP-ACP and PAFCP: 6.5% PA + fluoride varnish + CCP-ACP. All specimens were subjected to Vickers microhardness test (500 g, 10 seconds, 3 points). Data were analyzed using one-way ANOVA and Tukey’s post hoc test. The significance level was set at 0.05.

**Results:**

The mean and standard deviation (SD) values of Vickers hardness number (VHN) in groups C, PA, F, CP, PAF, PACP, FCP and PAFCP were 37.39±4.97, 38.68±4.62, 48.28±2.68, 41.91±3.32, 48.59±2.55, 53.34±2.57, 48.413±4.00 and 55.20±1.82, respectively. Pairwise comparisons of the groups revealed that there was no significant difference between groups C and PA, PA and CP, F and PAF, F and FCP, PAF and FCP, and PACP and FPACP (P>0.05); but significant differences were observed between other groups (P<0.05).

**Conclusions:**

The results of this study showed that the tested dentin treatments increased the microhardness of demineralized root dentin except for PA application.

## INTRODUCTION

With an increase in number of individuals preserving their natural teeth until old age, the challenge of oral healthcare provision for the aging population is becoming more significant. An increase in exposed root surface area in individuals older than 65 years of age increases the risk of root caries in them compared to younger populations [1]. Several studies have been performed on enamel remineralization; however, dentin remineralization is more challenging because dentin has lower mineral content and subsequently lower hardness and higher modulus of elasticity compared to enamel, which affect its properties [2,3]. Considering the microstructure of dentin, collagen fibers in dentin serve as a scaffold for mineral crystals that strengthen the matrix. Mineralized dentine matrix plays an important role in preventing crack propagation and thus maintaining the function of tooth. Therefore, it can be stated that remineralization of carious dentin restores the functionality of dentine [4]. Nowadays, preventive and minimally invasive dentistry offers various techniques to detect and restore minimal changes in tooth structure [5].

Fluoride is known as a remineralizing agent, which interacts with oral fluids on the enamel surface and subsurface and bonds to calcium and phosphate ions to form fluorapatite [6]. It has been reported to efficiently prevent enamel and dentin caries, and to arrest initial carious lesions in children and adolescents [7].

Casein phosphopeptide amorphous calcium phosphate (CPP-ACP) is a recently introduced remineralizing agent. This nanocomplex, derived from milk protein, was introduced as a supplemental source of calcium and phosphate ions [8]. The favorable effects of CPP-ACP are related to its ability to keep supersaturated levels of calcium and phosphate ions in the oral cavity when applied on the tooth surface [9,10].

The effect of natural products with antibacterial and remineralizing properties has been previously investigated on root dentin caries [11,12]. Grape seed or Vitis vinifera extract (GSE) contains flavonoids. Its other active ingredients include proanthocyanidin (PA), flavan-3-ols and catechin [13]. Studies have shown that glutaraldehyde and extracts rich in PA can be efficient for improving the mechanical stability of dentin and preventing collagen degradation [14,15]. In addition, PA increases the synthesis of collagen, promotes conversion of insoluble collagen to soluble collagen during development and decreases the rate of enzymatic degradation of collagen matrix [16].

Due to the lack of comprehensive studies comparing preventive approaches for root caries, dentin surface change in order to confer resistance against dental caries may serve as a novel modality in this respect. The objective of this study was to evaluate the effects of dentin biomodification using PA-rich GSE, fluoride varnish and CPP-ACP individually and in combination with each other on demineralization of root dentin in vitro using microhardness test. The null hypothesis was that there would be no significant difference between microhardness of the control group and the groups treated with remineralizing agents.

## MATERIALS AND METHODS

This study was approved by the ethical committee of the Vice Chancellor of Research, Hamadan University of Medical Sciences.

### Specimen preparation:

One-hundred and twenty freshly extracted sound human premolars without stains, morphological changes or cracks were used in this study. They were completely cleaned of organic debris and stored in 0.5% chloramine solution for 24 hours and then immersed in distilled water (grade 3, ISO 3696). The roots were cut from the crowns at the cementoenamel junction with a high-speed hand piece and diamond bur (Isomet 1000, Buehler, Lake Bluff, IL, USA). Four-millimeter-thick slices were cut out of the cervical third of the roots.

The root surfaces were polished under running water with 1,000-grit silicon carbide paper to remove cementum. A total of 120 slices were obtained and sealed with acid-resistant nail varnish (Revlon Corp., New York, NY, USA) except for a 3х4 mm window. The root sections of each tooth were horizontally mounted in self-cure acrylic resin (Aropars, Marlic Dental, Tehran, Iran) in prefabricated molds such that the buccal root surface remained exposed.

### The pH cycling and treatment:

The specimens were then subjected to demineralization by pH cycling. Each group was individually immersed in 500 mL of demineralizing solution (2.2 mM CaCl_2_, 2.2 mM KH_2_PO_4_, 50 mM acetic acid, pH of 4.3) for eight hours, immersed in distilled water for one hour, dried with absorbent paper and immersed in 500 mL of remineralizing solution (20 mM HEPES, 2.25 mM CaCl_2_, 1.35 mM KH_2_PO_4_, 130 mM KCl, pH of 7.0) for 15 hours. The treatment/pH cycling was continued for 15 days and all the solutions were made fresh and changed daily [17].

After 15 days, the root sections were rinsed with distilled water and dried. The specimens were randomly assigned to eight groups (n=15) for the following treatments:

Group C (no treatment), group PA, group F, group CP, group PAF, group PACP, group FCP and group PAFCP.

In group C, the teeth were rinsed with deionized water, blotted dry with absorbent paper and received no dentin treatment [18]. For PA solution preparation, powdered GSE was added to deionized water to a concentration of 6.5%. In group PA, PA solution was applied with a microbrush on the surfaces of specimens for two minutes. PA was rinsed off with deionized water for 10 seconds, and the teeth were blotted dry with an absorbent paper [19,20].

In group F, 5% (22600 ppm) fluoride varnish (Duraphat; Colgate-Palmolive, Piscataway, NJ, USA) was applied with a microbrush; varnish was left on the surface of the samples for one minute according to the manufacturer’s instructions [21]. In group CP, specimens were covered with CPP-ACP paste (GC Tooth Mousse, GC, Tokyo, Japan) for five minutes according to the manufacturer’s instructions [17]. In group PAF, the PA solution was applied with a microbrush on the surface of specimens for two minutes. PA was rinsed off and the teeth were blotted dry with an absorbent paper. After that, fluoride varnish was applied with a microbrush; varnish was left on the surface of the samples for one minute. In group PACP, PA solution was applied with a microbrush on the surface of specimens for two minutes. PA was rinsed off and the teeth were blotted dry with an absorbent paper [19,20]. After that, specimens were covered with CPP-ACP paste for 5 minutes and then, it was rinsed off and the teeth were blotted dry with an absorbent paper.

In group FCP, specimens were covered with CPP-ACP paste for five minutes and then, it was rinsed off and the teeth were blotted dry with an absorbent paper. After that, fluoride varnish was applied with a microbrush; varnish was left on the surface of the samples for one minute.

In group PAFCP, PA solution was applied with a microbrush on the surfaces of specimens for two minutes. PA was rinsed off and the teeth were blotted dry with an absorbent paper. After that, specimens were covered with CPP-ACP paste for five minutes and then, they were rinsed off and the teeth were blotted dry with an absorbent paper. Fluoride varnish was then applied with a microbrush; varnish was left on the surface of the samples for one minute. The specimens were finally placed in a container containing artificial saliva (Hypozalix, Biocodex, France). The main ingredients of artificial saliva were potassium chloride, sodium chloride, magnesium chloride, calcium chloride, dipotassium phosphate and monopotassium phosphate.

### Microhardness test:

All specimens were subjected to Vickers hardness tester (Micrometer, Buehler, Lake Bluff, IL, USA). The measurements were made at three different points for all samples. The load was 500 g for 10 seconds at room temperature. The mean surface microhardness in three points of all specimens was recorded as Vickers hardness number (VHN) in kgf/mm^2^.

### Statistical analysis:

The means and standard deviations (SDs) of microhardness were calculated. SPSS software version18 (SPSS Inc., IL, USA) was used to analyze the data via one-way ANOVA and Tukey’s test. The confidence level was set at 95% (α =0.05).

## RESULTS

The lowest mean VHN was observed in group C (37.39±4.97) and the highest in group PAFCP (55.20±1.82). Table 1 shows the mean and SD values of VHN in the eight groups.

**Table 1:** The microhardness values (VHN) in the groups

**Groups**	**Mean±SD**	**Minimum**	**Maximum**
C (control)	37.39±4.97^a^	31.20	45.10
PA	38.68±4.62^ac^	33.00	48.90
F	48.28±2.68^bf^	43.40	50.90
CP	41.91±3.32^c^	37.70	45.90
PAF	48.59±2.55^bdf^	45.00	52.00
PACP	53.34±2.57^ef^	50.50	58.70
FCP	48.41±4.00^bd^	41.80	54.60
PAFCP	55.20±1.82^e^	51.60	58.20

* Similar letters represent no significant difference; SD: Standard deviation

One-way ANOVA showed significant differences among the groups (P<0.001). Pairwise comparisons of the groups revealed no significant differences between groups C and PA (P=0.972), PA and CP (P= 0.189), F and PAF (P=1.000), F and FCP (P=1.000), PAF and FCP (P=1.000), and PACP and PAFCP (P= 0.826); but significant differences were found between other groups (P<0.05; Table 1).

## DISCUSSION

The prevalence of root caries has increased in the recent years due to increased life expectancy. Also, number of older people retaining their natural teeth has increased due to improved dental care. However, the teeth often have exposed root dentin due to gingival recession in older individuals [22]. Dentin remineralization is more complex and less effective than enamel remineralization because enamel has higher content of mineral crystals compared to dentin [23]. The concept of minimally invasive dentistry via remineralization of demineralized tooth structure is of great significance to preserve the remaining tooth structure [24,25]. Although numerous studies have shown the remineralizing potential of different agents, there have been limited comparative studies on the efficacy of fluoride varnish, CPP-ACP and PA solution mineralizing agents. It should be noted that PA has a chelating mechanism with calcium ions, which enhances mineral deposition on the surface of dentin [26]. When a higher concentration is used, PA needs to be delivered in a controlled manner with an additional external source of calcium and phosphate ions. In the current study, an in vitro model was used to evaluate and compare the remineralizing potential of fluoride varnish, CPP-ACP paste and 6.5% PA solution.

The in vitro pH-cycling models are used to simulate the dynamic range of deposition of minerals occurring in the natural process of development of carious lesions [12]. Panich and Poolthong [27] reported that the beneficial effects of CPP-ACP on microhardness of demineralized enamel increased in artificial saliva. Thus, for simulation of oral environment, the specimens were kept in artificial saliva in our study. The GSE used in this study is mainly composed of PA, which is a powerful antioxidant with known vasodilation and anti-inflammatory, anti-bacterial and anti-cancer effects [28]. Several studies have shown that GSE has remineralizing properties as well [12, 14, 15]. GSE has a high PA content. The PA-treated collagen matrices are non-toxic and inhibit the enzymatic activity of glucosyltransferase, F-ATPase and amylase glucosyltransferases, which are produced by Streptococcus mutans and polymerize the glucosyl moiety from sucrose and starch carbohydrates into glucans. This is the basis of the sucrose-dependent pathway of Streptococcus mutans and is critical for plaque formation and development of caries. Also, the adherent glucan contributes to the formation of dental plaque, accumulation of acids and subsequently localized decalcification of the enamel surface by facilitating bacterial adherence to the tooth surfaces, inter-bacterial adhesion and accumulation of biofilm. Therefore, inhibition of glucosyltransferases by PA can prevent dental caries [12,29,30].

In the current study, the microhardness values of group F versus C and CP, CP versus FCP and PAF, and also PA versus PAF and F were significantly different, while the difference in this respect between the groups CP and PA was not significant. These results were in agreement with those of Chokshi et al [6]. They showed that among the groups tested, fluoride varnish was the most effective remineralizing agent followed by CPP-ACP paste and FTCP. TCP is a new hybrid material produced with the milling technique that fuses beta tricalcium phosphate (β-TCP) and sodium lauryl sulfate or fumaric acid. This blending results in “functionalized” calcium and “free” phosphate, designed to increase the efficacy of fluoride remineralization [31,32]. Also, Shirahatti et al. [33] concluded that fluoridated dentifrices have substantial protective effect against caries formation; however, CPP-ACP paste did not have any additional influence on reducing the progression of lesions and its effect was similar to that of non-fluoridated dentifrices. Fluoride is believed to prevent dental caries through several mechanisms. One of them is formation of a calcium fluoride (CaF_2_)-like layer on the tooth surface enhancing deposition of minerals such as fluorapatite or fluorohydroxyapatite [34]. It also decreases acid production by microorganisms, inhibits intracellular and extracellular enzymes, and replaces hydroxide ions [21]. It should be noted that, the microhardness values of group F versus PAF and FCP were not significantly different in this respect, and no considerable increase was observed. One explanation is that the fluoride varnish application has a dominant effect to increase microhardness of the study groups. Fluoride varnish was applied as the final remineralization agent and it was not rinsed off and was allowed to dry. Thus, fluoride formed a superficial layer on the surface. On the other hand, microhardness values of groups PACP and PAFCP were not significantly different in our study. Although the fluoride ions increase tooth surface resistance to demineralization, the resulting remineralization, despite its advantages, is a self-limiting phenomenon, which prevents penetration of calcium and phosphate ions into deeper layers [35]. Nevertheless, fluorosis and toxicity in high dose are the side effects of fluoride. Thus, efforts to find effective cariostatic compounds with minimal adverse effects are ongoing [36].

In the present study, the degree of remineralization (VHN) of group CP versus C, PACP versus PA and PAFCP versus PAF was significantly different. This finding was similar to the results of studies performed by Lata et al, [35] and Pulido et al, [37] suggesting that treatment with CCP-ACP significantly increases tooth surface microhardness.

The CPP includes peptides that are derived from milk protein (casein) forming complexes with calcium and phosphate. The CPP contains a cluster of phosphoseryl residues that stabilize nanoclusters of ACP in metastable solution. The CPP binds to surfaces such as plaque, bacteria, soft tissue and dentin (owing to its sticky nature), providing a reservoir of bioavailable calcium and phosphate in the saliva and on the surface of teeth [6]. It can diffuse into the porous lesions and penetrate deep into the demineralized lesions [35]. CCP-ACP has an action similar to that of fluoride to inhibit cariogenic bacteria and demineralization and enhance remineralization. It has been further reported that in contrast to fluoride that mostly remineralizes superficial areas of the lesion, CPP-ACP can remineralize deeper areas of the lesion due to its smaller molecular size [38, 39].

In the present study, the mean microhardness values of group CP versus PACP and FCP versus FPACP were significantly different. The PA is capable of increasing the number of collagen cross-links in dentin, resulting in improved mechanical properties of tooth [14,40]. Shi et al. [41] showed that the PA positively affects the remineralization processes of artificial dentinal carious lesions, and may be a promising natural agent for remineralization therapy instead of fluoride. This finding was confirmed by Benjamin et al [42]. Additionally, PA is acidic and pH of CPP-ACP paste may be reduced by the addition of PA [26]. This would enhance remineralization by releasing more amorphous calcium phosphate ions into the carious lesion. On the other hand, the calcium-binding effect of PA may also allow greater mineral deposition within the carious lesion.

Despite the slight increase in microhardness following the application of PA, there was no statistically significant difference between the group PA versus CG and F versus PAF in this regard. The remineralizing effect of PA appears to be distinct from the action of fluoride [28]. A possibility is that the behavior of PA molecules may have played a role. Effect of PA may be restricted to the superficial layer due to high molecular weight of PA molecules and that it cannot penetrate deep into the underlying layers [43]. The current findings were in accord with those of Arumugum et al, [44] and Broyles et al [19]. In fact, PA contributed to mineral deposition on the lesion surface only, which inhibited further mineral deposition in the deeper part of the lesion [12].

Similar to the current study, Shi et al. [41] showed no statistically significant difference between groups NaF versus GSE + NaF. Further studies are needed to identify the active constituents of GSE and maximize its effect on the substrate. Combined use of PA, CPP-ACP and fluoride varnish in PAFCP group had a synergistic effect on remineralization of root caries as well as optimal interaction of the minerals with the collagen matrix, because of regaining some part of the mechanical characteristics of the collagen matrix [45]. The microhardness value in this group was higher than that in other groups. This finding was in agreement with the results of Epasinghe et al [46]. In combined groups of this study, the significant difference between the microhardness values of groups FCP and PAFCP may be due to the presence of PA that leads to small increase in microhardness. The suppressing effect of PA on enzymes, such as collagenase, is an added benefit in stabilizing the collagen matrix. Apart from the collagen matrix, PA may also bond to non-collagenous proteins. Some proteins may play a role in mineral deposition in dentin structure [47]. However, future clinical studies are recommended since remineralization in vitro may be largely different from the dynamic biological system in vivo. On the other hand, nanomechanical testing is necessary to understand the mechanical recovery of remineralized dentin at the nanostructural level. It should be noted that improved remineralizing methods are required to arrest the process of dental caries, particularly in individuals at high risk of caries, and future studies are recommended in this field.

## CONCLUSION

Within the limitations of this study, it can be concluded that all remineralizing agents successfully caused remineralization of artificial carious lesions after treatment, except for PA solution in group PA. Also, group PAFCP showed the highest remineralization potential followed by PACP.
